# Dual-degenerate TCRs target multiple KRAS hotspot and HLA-A3 family combinations

**DOI:** 10.21203/rs.3.rs-10030408/v1

**Published:** 2026-06-19

**Authors:** Minying Zhang, Barbara Nassif Rausseo, Peixin Jiang, Emily Bontekoe, Amanda Montoya Alves, Jared Slone, Waree Rinsurongkawong, Vadeerat Rinsurongkawong, Anika Patel, Jeff Lewis, Michael Davies, Patrick Hwu, Jack Lee, Ignacio Wistuba, Ara Vaporciyan, Drew Deniger, Greg Lizee, Cassian Yee, Lydia Kavraki, Don Gibbons, Hai Tran, Jianjun Zhang, John Heymach, Alexandre Reuben

**Affiliations:** The University of Texas MD Anderson Cancer Center; The University of Texas MD Anderson Cancer Center; The University of Texas MD Anderson Cancer Center; The University of Texas MD Anderson Cancer Center; The University of Texas MD Anderson Cancer Center; Rice University; The University of Texas MD Anderson Cancer Center; The University of Texas MD Anderson Cancer Center; Rice University; The University of Texas MD Anderson Cancer Center; The University of Texas MD Anderson Cancer Center; Moffitt Cancer Center; The University of Texas MD Anderson Cancer Center; Moffitt Cancer Center; The University of Texas MD Anderson Cancer Center; The University of Texas MD Anderson Cancer Center; The University of Texas MD Anderson Cancer Center; The University of Texas MD Anderson Cancer Center; Rice University; The University of Texas MD Anderson Cancer Center; The University of Texas MD Anderson Cancer Center; The University of Texas MD Anderson Cancer Center; The University of Texas MD Anderson Cancer Center; The University of Texas MD Anderson Cancer Center

## Abstract

Oncogenic KRAS mutations drive a substantial proportion of lung cancers and are linked to poor prognosis, positioning KRAS as a compelling target for cellular immunotherapy. Thirty-five percent of lung adenocarcinomas harbor the KRAS G12C, G12V, G12D mutations. Here, we developed HLA-A*03:01- and HLA-A*11:01-restricted T cell receptors (TCR) targeting the most prevalent G12C and G12V KRAS hotspot mutations in lung adenocarcinoma. Predicted high affinity peptides were screened using our TCR discovery and validation pipeline, and functional assessment was performed to determine sensitivity, specificity, and cytotoxic potential of TCR-engineered T cells. We discovered and validated 5 novel TCRs targeting KRAS G12C and G12V 9-mers, each of which demonstrated an ability to recognize and lyse tumor cells endogenously presenting mutant KRAS on HLA-A*03:01 or HLA-A*11:01. Notably, several TCRs demonstrated distinct modes of cross-reactivity, including peptide degeneracy across KRAS G12 variants, HLA degeneracy across HLA-A*03:01 and HLA-A*11:01, or dual degeneracy across both KRAS and HLA, thereby broadening the treatable target populations. TCRs that recognize the KRAS hotspot shared sequence motifs were found in several lung cancer patients. Our study highlights the successful generation of multi-valent KRAS-specific TCRs and supports the feasibility of targeting shared KRAS neoantigens through TCR engineering in lung cancer.

## Introduction

Lung cancer remains the leading cause of cancer-related mortality in the US, accounting for 20% of all cancer deaths in 2024. Lung adenocarcinomas (LUAD) represent about 40% of all lung cancers, among which 35% harbor mutations in the Kirsten rat sarcoma viral oncogene homolog (KRAS), primarily G12C, G12V, and G12D. KRAS mutations are also observed in other adenocarcinomas of the colon (COAD: 40–50%) and pancreas (PAAD: 80–90%) (1), and drive oncogenesis by constitutively activating downstream signaling pathways, including MAPK and PI3K-AKT (2, 3). Despite their success, therapeutic resistance to the KRAS G12C inhibitors sotorasib and adagrasib demands alternative strategies for targeting KRAS-driven lung cancer (4).

Advances in immunotherapy over the past decade have revolutionized cancer treatment, offering the potential for highly specific and durable responses. Cellular immunotherapy involving engineering of the T cell receptor (TCR) into T cells (TCR-T) represents a promising approach to target tumor-restricted neoantigens (5). Unlike chimeric antigen receptor (CAR) T cell therapy which is limited to surfaceexpressed proteins, TCR-T can recognize epitopes derived from intracellular proteins processed and presented on HLA molecules. Here, we describe and validate multiple TCRs against HLA-A*03:01- and HLA-A*11:01-restricted epitopes of mutant KRAS.

## Methods

### Clinical Data

The GEMINI cohort includes 1,380 unique lung adenocarcinoma patients with KRAS mutations. KRAS and HLA status was determined through sequencing at the American Red Cross, Ziopharm Oncology/Alaunos Therapeutics, or AdaptiveImmune. All human tissues were obtained through The University of Texas MD Anderson Cancer Center’s approved Institutional Review Board (IRB) protocol (GEMINI: PA13–0589, PA16–0062; ICON: PA15–1112; PROSPECT: LAB07–0233).

### Cell lines

H1792, H441, H1650, H2444 and HEK-293 cell lines were obtained from ATCC. Target tumor cell lines were constructed by transducing the restricting HLA-A*03:01 or HLA-A*11:01 allele and KRAS mutations pENTER vector by GenScript USA, Inc. (Piscataway, NJ), when required. Final HLA allele constructs were created by Gateway^™^ LR Clonase^™^ II Enzyme Mix (Invitrogen^™^, 11791020) into the lentiviral vector pLV401GFP (6). Tumor cells successfully transduced with HLA alleles were sorted based on GFP expression. KRAS mutant antigen was cloned into PHAGE vector (Addgene, 24526), isolated by puromycin selection, and overexpressed in tumor cell lines expressing the desired HLA.

### KRAS mutant neoantigen predictions

Predicted KRAS mutant epitopes were identified using NetMHCpan4.1 (7). Epitopes were selected based on their predicted ability to strongly bind (% Rank < 0.5) to the 10 most prevalent HLA class I alleles in the United States (8). Peptides were synthesized by Genemed Synthesis, Inc. (San Antonio, TX) and GenScript USA, Inc. (Piscataway, NJ) with a purity > 95%.

### Dendritic cell maturation

Healthy donor leukaphereses were purchased from Charles River Laboratories (San Francisco, CA) and All Cells (Alameda, CA) for PBMC isolation by Ficoll gradient centrifugation. Monocytes from healthy donor PBMC were cultured for one week in AIM-V medium (Gibco, 12055–083) supplemented with 800U/ml of recombinant human GM-CSF (ThermoFisher, 215-GM) and 500U/ml of recombinant human IL-4 (R&D, 204-IL-050) to generate dendritic cells (DCs). DCs were matured with 10ng/ml of recombinant human TNFγ (R&D, 210-TA), 2ng/ml of recombinant human IL-1β (R&D, 201-LB-005), 1000U/ml of recombinant human IL-6 (R&D, 206-IL-010), and 1000ng/ml of Prostaglandin E2 (MP Biomedicals, 219457601). Following maturation, DCs were pulsed with 10μM of peptide for 4 hours at room temperature and irradiated at 5000 rad.

### Antigen-specific T cell stimulation

Antigen-specific T cells were generated using autologous PBMC mixed with irradiated and peptide-pulsed DCs at 35:1 ratio cultured in T-cell medium (LymphoONE T-Cell Expansion Xeno-Free Medium, Takara, WK552S) supplemented with 5ng/ml of recombinant human IL-7 (R&D, 207-IL-005), and 30ng/ml of recombinant human IL-21 (PeproTech, AF-200–21) for one week to enhance growth of antigen-specific T cells. Cultured T cells were re-stimulated with irradiated, peptide-pulsed DCs as described above. Recombinant human IL-2 at 10U/ml was supplemented every two days.

### Isolation of antigen-specific T cells

HLA tetramers were synthesized by the Baylor MHC Tetramer Production Core (Houston, TX) or the Fred Hutchinson Cancer Research Center Tetramer Core (Seattle, WA). Antigen-specific T cells were stained with PE-conjugated tetramer and PB- or BV421-conjugated anti-CD8 (BD Biosciences Pharmingen, 558207 or 562428) and sorted for tetramer/CD8 double-positive T cells using BD FACSAria III. The sorted antigen-specific T cell population was expanded using a Rapid Expansion Protocol (REP) (9). Expanded antigen-specific T cells were further purified by repeating flow cytometric cell sorting for tetramer/CD8 double-positive T cells.

### T cell cytotoxicity assays

Cytotoxic potential of T cells was assessed by ^51^Cr release assay (10). Briefly, target cells in appropriate growth medium (lung cancer cell lines: RPMI Gibco, 11875–0930; 293: DMEM Corning, 10–013-CV) supplemented with 10% fetal bovine serum (GenDEPOT, F0601–050) containing 100μl of ^51^Cr were incubated at 37°C for 2 hours. Target cells were washed to remove free chromium and seeded in 96-well U bottom cell culture plates. Effector T cells were added at varying ratios. Supernatant was periodically collected between 4 to 24 hours of incubation and chromium released by lysed target tumor cells was measured by 2450 Microbeta2 microplate counter (PerkinElmer).

### Identification of TCRα/β chains

Expanded antigen-specific T cells were used for library construction with 10X Genomics Single Cell VDJ 5’ Kit at MD Anderson Advanced Technology Genomics Core. RNA and TCR libraries were sequenced using NextSeq 500 and reads were processed with Cell Ranger 5.0.0. Paired TCR α and β chains were linked through their shared barcode.

### Recombinant TCR retroviral production

Full length TCRα/β genes were synthesized by GenScript USA, Inc. (Piscataway, NJ) and cloned into the pMSGV1 retroviral vector as previously described (11). Briefly, TCR constructs were designed with Furin and P2A cleavage to connect TCRα and TCRβ chains for maximum release of TCRα/β chains after expression. Viral particles were generated with pMSGV1-TCR construct and RD114 envelope construct (12) by co-transfection into the retroviral packaging cell line GP2–293 (Takara Bio, 631458) with jetPRIME transfection reagent (Polyplus, 101000015). Following 48 hours of transfection, supernatant was collected and used for downstream T cell transduction.

### Generation of TCR-engineered T cells

Healthy donor PBMCs were used to isolate CD8^+^ T cells by negative selection using Human CD8^+^ T cell Isolation kit (Stemcell, 17953). Isolated CD8^+^ T cells were activated by culturing T cells in T-cell medium (LymphoOne^™^ T cell expansion medium, Takara, WK552S) supplemented with 100ng/ml of anti-CD3 antibody (clone OKT3, Miltenyi Biotec, GMP CD3) and 50U/ml of recombinant human IL-2 for 48 hours. 24-well non-tissue culture treated plates were coated with 20μg/ml of RetroNectin (Takara, T110B) according to the manufacturer’s protocol. Supernatant collected from the retroviral transfection containing TCR viral particles was added to RetroNectin-coated plates and centrifuged at 2000*g* for 2 hours at 32°C for TCR-T cell transduction. Unbound viral particles were removed from the 24-well plate. Next, 2×10^5^ activated CD8^+^ T cells were added to each well and incubated for 5 days in T-cell medium supplemented with 50U/ml of human recombinant IL-2. Virally-transduced TCR-T cells were stained for CD8^+^/ tetramer staining and isolated by flow cytometry sorting using BD FACSAria III.

### Identification of functional residues and cross-reactivity assessment

Alanine scanning was utilized to identify amino acid residues vital in antigen binding of the T cells. For both alanine scanning and cross-reactivity assessments, 293 cells were incubated with individual peptides at 10μM of final concentration for 4 hours at 37°C and washed once with T-cell medium. T cells were co-cultured with peptide-pulsed 293 cells at a 5:1 effector overnight at 37°C. To determine the reactivity of T cells towards peptides, secretion of MIP-1β was measured by Duo set Human MIP-1β Instant ELISA kit (Invitrogen, BMS2030INST) according to the manufacturer’s instructions. ELISA plates were read with SmartReader^™^ 96 Absorbance Plate Reader (Accuris, MR9600). Concentration of MIP-1β for samples was calculated according to the standard curve using GraphPad Prism version 10.3.1 for Windows (GraphPad, USA).

### Grouping of lymphocyte interactions by paratope hotspots

To detect TCRs related to validated KRAS-specific TCRs, GLIPH2 (13) was used to analyze TCRβ CDR3 sequences from ICON and PROSPECT and sequences were grouped based on their purported shared antigen specificities and motif similarities. The clustering output from GLIPH2 was visualized using igraph.

### Modeling pHLA Complex Structures

3D structural models for peptide-MHC complexes were generated using the tool APE-Gen 2.0 (14). APE-Gen 2.0 was run with automatic anchor selection, default anchor tolerance, and the default number of random coordinate descent steps. The receptor/MHC side chains were treated as flexible during docking and optimization. Energy minimization was carried out with OpenMM (15). The peptide poses with the best score according to Vinardo (16) were selected for further analysis and are shown in Fig. 4A. Comparative Coulombic electrostatic potential maps were generated via ChimeraX (17) for residues that differed among the pMHCs evaluated in this work.

### Statistical analysis

All statistical analyses were performed using GraphPad Prism v10.3.1. Unpaired t-tests were performed when appropriate based on data types and statistical assumptions. A p-value of < 0.05 was considered statistically significant. Plots were generated using either FlowJo v10, GraphPad Prism or Python package matplotlib.

## Results

### KRAS mutation profiles and HLA-A subtype prevalence in lung adenocarcinoma patients

**Figure 1:**
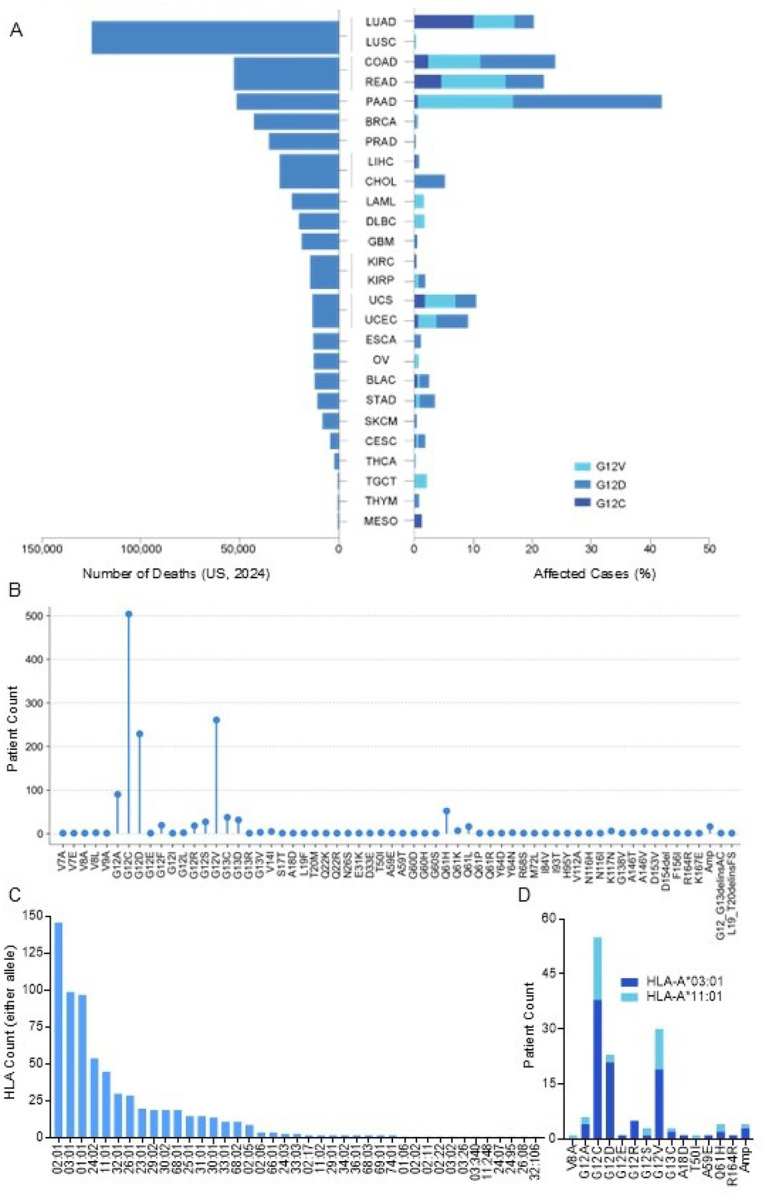
KRAS mutations in residue 12 are highly prevalent in lung adenocarcinoma. **A)** Left: Number of cancer deaths across tumor histologies from public databases (38–40). Right KRAS mutation prevalence across KRAS^G12C/V/D^ in TCGA cohorts (41). **B)** Number of LUAD patients with KRAS mutated at each residue in GEMINI cohort (n=1,380). **C)** HLA-A count for either allele for all KRAS mutant LUAD patients in GEMINI (n=694). **D)** Proportion of KRAS mutations for only LUAD patients expressing HLA-A*03:01 or HLA-A*11:01 in GEMINI.

To establish the prevalence of cancer patients affiicted by KRAS^G12C/V/D^ mutations, we evaluated all affected TCGA cohorts using NCI’s Genomic Data Commons. LUAD (20%, 113/559 patients) had the most cases of KRAS^G12C/V/D^ combined, followed by COAD (24%, 102 / 428 patients) and PAAD (42%, 75 / 179 patients) (Fig. 1A). G12C (10%) and G12V (7%) were more prevalent in LUAD than G12D (3%) compared to other cancers with higher G12D-affected cases (54 COAD, 45 PAAD). We further examined the distribution of KRAS^G12C/V/D^ in LUAD patients at MD Anderson Cancer Center by leveraging GEMINI (n = 7,737 LUAD) and identified 1,380 patients (18%) harboring KRAS mutations among which the four most prevalent were G12C (504 patients, 37%), G12V (261 patients, 19%), G12D (229 patients, 17%), and G12A (90 patients, 7%), consistent with prior reports (Fig. 1B) (18–20).

To identify immunogenic epitopes, we predicted the highest potential KRAS G12^C/V/D^-derived epitopes with NetMHCpan-4.1 using the top 10 HLA alleles in the U.S. (7, 8). HLA-A*03:01 was selected due to having the highest predicted binding affinity for all three KRAS 9-mers (*VVGA*C*GVGK*, *VVGA*V*GVGK*, *VVGA*D*GVGK*) (Table 1). HLA-A*11:01 was also included as a prevalent member of the A3 superfamily with similar binding motif (21). In GEMINI, HLA-A*02:01 (21.0%), HLA-A*03:01 (14.3%), and HLA-A*01:01 (13.9%) were the three most prevalent HLA-A alleles in KRAS-mutant LUAD (Fig. 1C), consistent with their nation-wide prevalence (8), while HLA-A*11:01 (6.5%) was the 5th most prevalent. KRAS G12C, G12V, and G12D were the three most prevalent KRAS alterations among patients expressing HLA-A*03:01 and HLA-A*11:01 (Fig. 1D).

### KRAS G12C and G12V 9-mers are immunogenic when presented on HLA-A*03:01 and HLA-A*11:01

We used our established pipeline for the discovery and validation of TCRs from peripheral blood (**Supplementary Fig. 1**) (22). In short, PBMCs from four healthy donors were used to identify naturally-occurring TCRs that recognize each epitope of interest. Following stimulation with peptide-pulsed dendritic cells (DCs), rare populations (< 0.5%) of antigen-specific peripheral T cells were identified using tetramer-guided cell sorting and rapid expansion protocols (REP) (**Supplementary Fig. 2A**). Antigen-specific T cells were subsequently isolated and expanded via REP, as previously described (9) (**Supplementary Fig. 2A**), although KRAS^G12D^-specific T cells were not detected using our pipeline. To confirm the identified antigen-specific T cell recognized KRAS epitopes that were endogenously-processed and presented, we used the LUAD cell lines H1792^G12C^ and H441^G12V^ which were transduced with the relevant HLA. In absence of a lung cancer cell line, 293^WT^ were transduced with KRAS mutants and restricting HLAs (Table 2). Tumor cell lines were then co-cultured with antigen-stimulated T cells and cytotoxicity was assessed by ^51^Cr release. As illustrated in **Supplementary Fig. 2B**, KRAS G12C-specific T cells lysed 100% of HLA-matched target cells pulsed with the G12C 9-mer. KRAS G12V-specific T cells lysed 80% of target cells pulsed with G12V 9-mer, compared to no lysis in unpulsed HLA-matched targets (*p < 0.0001*) (**Supplementary Fig. 2C**). Importantly, KRAS G12V-specific HLA-A*03:01-restricted T cells also lysed 15% of unpulsed HLA-matched target cells H441^G12V^ demonstrating clonotypes were capable of eliciting a response to endogenously-processed and presented epitopes (**Supplementary Fig. 2C**). A similar response was seen in G12C-specifc T cells, lysing 5–10% of unpulsed targets (**Supplementary Fig. 2B**).

### Functional validation of KRAS G12C- and G12V-specific TCR-T cells

**Figure 2:**
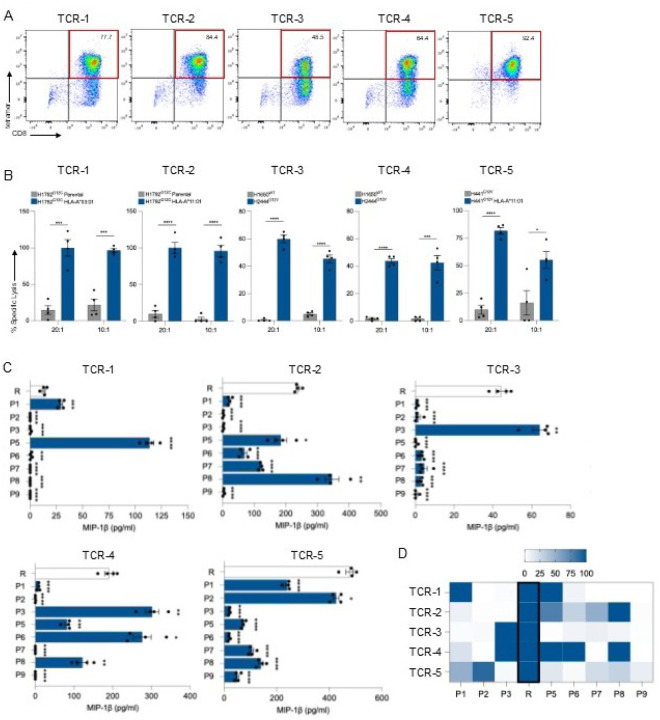
KRAS-specific TCRs retain cytotoxic potential when engineered into PBMCs. **A)** Tetramer staining of TCR-transduced HLA-matched donor CD8+ T cells following REP. **B)** ^51^Cr release assay of co-cultured TCR-1-transduced T cells with H1792^G12C^; TCR-2-transduced T cells with H1792^G12C^; TCR-3- and TCR-4-transduced cells with H2444^G12V^ or H1650^WT^; ^51^Cr release assay of co-cultured TCR-5-transduced T cells with H441^G12V^. **C)** MIP-1β production by TCR-T cells cocultured at a 5:1 E:T ratio with peptide-pulsed 293 cells presenting an alanine scan of the KRAS 9-mer epitope. Analyzed with Unpaired t test, bars represent mean value +/− SEM, each dot represents a technical replicate; P1–9 denotes position of alanine replacement, R denotes unmutated reference peptide which contains an alanine in position 4. **D)** Heatmap represents percent change in TCR-T cell recognition based on reference peptide after animo acid replacement. Four replicates were performed. *p<0.5, **p<0.01, ***p<0.001, ****p<0.0001.

To identify αβTCR pairs targeting specific KRAS mutations, we next performed single cell TCR/RNA sequencing. A total of 138 G12C/HLA-A*03:01, 247 G12C/HLA-A*11:01, 666 G12V/HLA-A*03:01, and 46 G12V/HLA-A*11:01 unique clonotypes were identified in each respective population. The single most dominant clonotype in each sample was found to comprise 62.7% TCR-1 (G12C/HLA-A*03:01), 56.8% TCR-2 (G12C/HLA-A*11:01), 41.8% TCR-3 (G12V/HLA-A*03:01), 16% TCR-4 (G12V/HLA-A*03:01), and 71.9% TCR-5 (G12V/HLA-A*11:01) of sorted cells. The most dominant TCRs were then synthesized and introduced into retroviral vectors to transduce CD8 T cells from HLA-matched healthy donor PBMCs, which were then sorted and expanded by REP. Transduction efficiency varied from 48–92% (Fig. 2A). TCR-T populations were purified via tetramer sorting and used to assess cytotoxic potential using H1792^G12C^, H2444^G12V^, and H441^G12V^ cells transduced with the appropriate HLA, as needed (Table 2).TCR-1 (*p = 7.5×10*^*− 6*^) and TCR-2 (*p = 2.7×10*^*− 6*^) lysed 90–100% of H1792^G12C^, only when the restricting HLA was expressed (Fig. 2B). TCR-3 (*p = 5.3×10*^*− 6*^) and TCR-4 (*p = 9.2×10*^*− 5*^) lysed 40–50% of HLA-matched target H2444^G12V^ with insignificant killing of irrelevant target control H1650^WT^(Fig. 2B). Lastly, TCR-5 lysed 82% of HLA-matched target H441^G12V^ at 20:1 (*p* = 0.125) and 55% of H441^G12V^ at 10:1 (*p* = 0.088), with 10% killing of HLA-untransduced H441^G12V^ which endogenously expresses HLA-A*03:01 (Fig. 2B). Overall, antigen-specific cytotoxic responses were noted for all five tested TCR-T. We next used alanine scanning to identify candidate TCR-engaging residues within each epitope. Consistent with previous studies, alanine at the known anchor residue positions 2 and 9 frequently abrogated T cell activation, as measured by secreted MIP-1β (23, 24) (Fig. 2C). Interestingly, replacing P2 with alanine resulted in only a modest decrease in TCR-5 activation (Fig. 2D), even though alanine is rarely found at P2 in peptides naturally presented by HLA-A*03:01 and HLA-A*11:01 (25). Interestingly, the reactivity of TCR-1 was enhanced 7-fold when residue 5 of the G12C 9-mer (VVGACGVGK) was mutated to an alanine (VVGAAGVGK), corresponding to the prevalent KRAS G12A hotspot mutation (Fig. 2D–E).

### Matched KRAS-specific TCR sequences arise naturally in lung cancer patients

**Figure 3:**
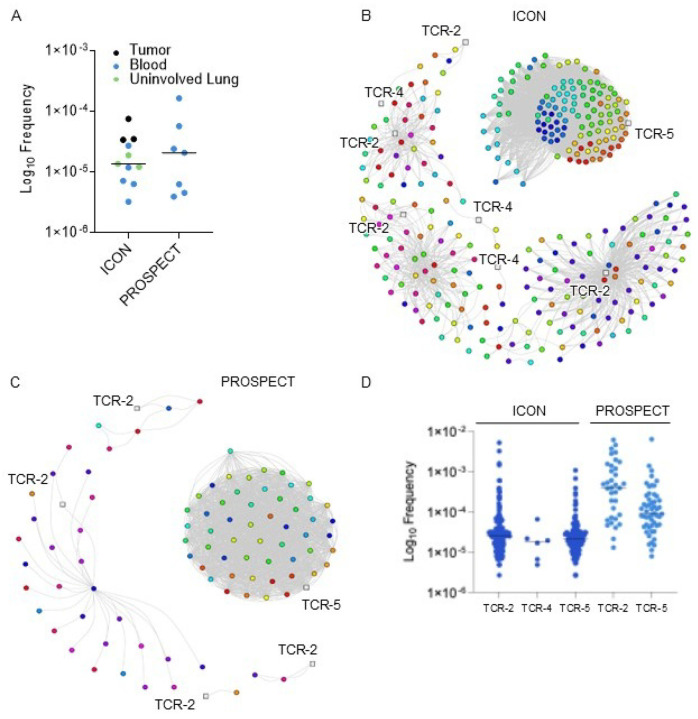
Frequency of matched KRAS-specific TCRs in lung cancer patients from ICON and PROSPECT cohorts. **A)** Log10 frequency of TCR-2-g12c sequence in samples from ICON and PROSPECT. Each dot represents a single patient within each cohort and color represent sample origin. GLIPH2 analysis of TCR relatedness between validated TCRs and TILs from **B)** ICON and **C)** PROSPECT. Each dot represents a single patient tumor sample. Relatedness is expressed as the hop distance from the patient’s TCR node to the node of the validated TCR sequence. **D)** Log10 frequency for TCRs with shared motifs in ICON and PROSPECT. Quantification representative of **B C)** cluster analysis. Each dot represents a patient TCR related to the validated TCR listed on x-axis. The horizontal line represents the median.

Because TCRs were initially generated from healthy donor blood, we next sought to determine whether they could be identified in lung cancer patients. To do so, bulk CDR3β sequencing was performed on resected tumors from ICON (n = 104) and PROSPECT (n = 204) to identify matching TCRs (26). Interestingly, TCR-2 was identified in 18 lung patients, although exact matches for the other CDR3s were not (Fig. 3A). Analyses were expanded to incorporate related TCRs using GLIPH2 (13) to cluster convergent TCR sequences. Several related TCRs clustered with our validated TCRs’ CDR3β sequences in both ICON (TCR-2, n = 65; TCR-4, n = 5; TCR-5, n = 57) and PROSPECT (TCR-2, n = 27; TCR-5, n = 44) (Fig. 3B–C). TCR-2 and TCR-5 had the highest number of related TCR incidences in both cohorts, while TCR-4 exhibited the least (Fig. 3D). These analyses confirm that these validated KRAS-specific TCRs and closely related TCRs arise naturally in lung cancer patients.

### Unique TCR degeneracy patterns emerge across HLAs and KRAS mutations

**Figure 4:**
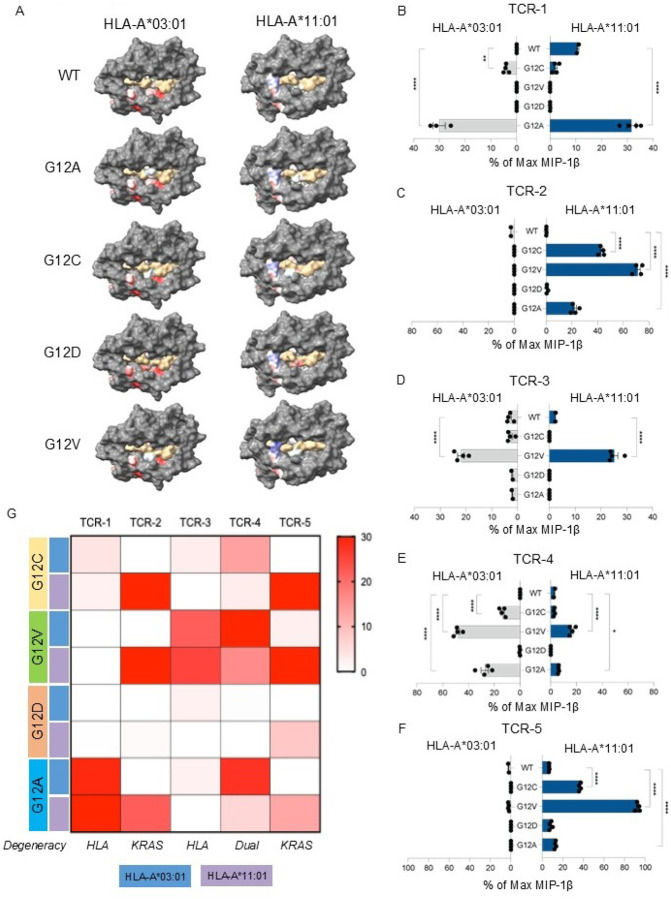
KRAS-mutations peptide degeneracy of identified TCRs across HLA-A03:01 and HLA-A11:01. **A)** Predicted 3D structures of the pHLA complexes examined in this study. All structures were modeled using APE-Gen 2.0. HLA residues shared between HLA-A*03:01 and HLA-A*11:01 are colored in gray. Similarly, residues conserved across the KRAS peptides are colored in yellow. Residues that differ are colored according to their charges, with blue being more positive and red being more negative. Measurement of MIP-1β production by ELISA from supernatant following 2 hour coculturing of 293-peptide pulsed cells (KRAS WT, G12C, G12V, G12D, or G12A 9-mer peptides with **B)** TCR-1, **C)** TCR-2, **D)** TCR-3, **E)** TCR-4, or **F)** TCR-5. Grey bars represent 293^WT^ HLA-A*03:01 cells and blue represents 293^WT^ HLA-A*11:01 cells pulsed with respective peptide and cocultured with respective TCR-T cells. Percent of maximum MIP-1β was calculated from maximum secretion obtained from PMA and Ionomycin treatment. Analyzed with One-way ANOVA multiple comparisons; bars represent mean value +/− SEM, each dot represents a technical replicate, four replicates performed. **G)** Heatmaps summarize TCR-T cell recognition of mutant 9-mer peptides presented by HLA-A alleles, derived from the data shown in Fig. 4B–F. *p<0.5, **p<0.01, ***p<0.001, ****p<0.0001

We next sought to further investigate potential TCR degeneracy on the basis of alanine scan results demonstrating conserved recognition of the KRAS G12A peptide on both HLA-A*03:01 and HLA-A*11:01. Prior studies have shown that a single TCR can cross-react with the same antigen presented by both HLA-A*03:01 and HLA-A*11:01 (22), likely because these alleles belong to the HLA-A3 superfamily and therefore share similar peptide-binding motifs and anchor residue preferences. However, cross-reactivity between these HLAs is not guaranteed. Figure 4A shows the predicted 3D structures of wild-type KRAS bound to HLA-A*03:01 and HLA-A*11:01 and highlights the amino acids that differ between both HLAs. Residues surrounding the groove of HLA-A*03:01 exhibit a more negative electrostatic potential, whereas the corresponding region in HLA-A*11:01 is more positively charged due in part to the presence of arginine at position 163 in place of threonine. These structural and electrostatic differences influence TCR specificity, and motivated further investigation into the extent of TCR degeneracy across HLAA*03:01 and HLA-A*11:01 (Fig. 4A).

In addition to potential cross-HLA reactivity, we sought to investigate TCR recognition across unique 9-mer epitopes arising from substitutions at the KRAS G12 position. Experimentally determined structures of multiple TCRs reactive against KRAS-G12V have shown that the CDR3β loop directly contacts position 5 of the peptide (27), suggesting that mutations at this position may strongly influence TCR recognition. The fact that our TCRs were reactive against the mutated KRAS antigens G12C and G12V, which present cystine and valine at position 5 respectively, but not the native peptide, which has a glycine at position 5, further suggests that mutations at position 5 of the peptide can have strong effects on TCR recognition. However, our alanine scan experiments demonstrated TCR recognition of the KRAS-G12A peptide, indicating that substitutions at peptide position 5 do not uniformly disrupt TCR binding. These findings motivated a broader investigation into TCR degeneracy across distinct KRAS G12 mutant epitopes.

To evaluate the breadth of antigen recognition and potential cross-HLA reactivity, we assessed the functional responses of the five KRAS-specific TCRs against a panel of KRAS G12 variants presented on HLA-A*03:01- and HLA-A*11:01-transduced 293 target cells. Among these, receptors illustrated distinct patterns of cross-reactivity including KRAS degeneracy, HLA degeneracy, and dual degeneracy (both KRAS and HLA degenerate). As shown in Fig. 4, TCR-1 and TCR-3 primarily demonstrated HLA degeneracy, maintaining recognition across both HLA-A*03:01 and HLA-A*11:01 for KRAS G12A and G12V, respectively. In contrast, TCR-2 and TCR-5 exhibited KRAS degeneracy while remaining restricted to HLA-A*11:01. Strikingly, TCR-4 displayed dual degeneracy, demonstrating an ability to recognize KRAS G12C, G12V, and G12A on HLA-A*03:01 while also recognizing KRAS G12V and G12A on HLA-A*11:01 (Fig. 4B–F).

TCR-2 exhibited broader peptide cross-reactivity within its restricting allele, recognizing KRAS G12C, G12V, and G12A peptides, but not WT or G12D (Fig. 4C& G). TCR-2 was found to have a 1.5-fold higher reactivity to G12V than to G12C peptide. However, this TCR failed to respond to the same peptide panel, including G12C cognate peptide, when presented on HLA-A*03:01, indicating strict HLA restriction despite KRAS degeneracy. In contrast, TCR-4 displayed the broadest cross-reactivity profile. In addition to its cognate KRAS G12V peptide, this receptor recognized G12C and G12A variants presented on HLA-A*03:01 (Fig. 4E & G). This pattern was maintained when peptides were presented on HLA-A*11:01, although overall reactivity was reduced by approximately 3-fold, suggesting partial tolerance to HLA mismatch with reduced functional avidity.

Structural and physicochemical differences between the two HLAs are primarily located around the N-terminus of the peptide (Fig. 4A) while amino acid composition around C-terminus of the peptide is largely conserved between HLA-A*03:01 and HLA-A*11:01. It appears that TCR-1, TCR-3, and TCR-4 are tolerant to these differences, while TCR-2 and TCR-5 are not. This difference could possibly be due to TCR specific binding modes, which may be shifted towards the N- or C-terminus of the peptide (28). Most importantly, all TCRs were also evaluated against the WT KRAS 9-mer peptide with little to no detectable response (Fig. 4B–F).

## Discussion

In this study, we discovered and validated TCRs recognizing endogenously-expressed lung cancer-associated mutant KRAS epitopes presented by HLA-A*03:01 and HLA-A*11:01 and found all TCRs presented KRAS epitope degeneracy, HLA degeneracy or both. Our findings support previous reports that HLA class I presents the KRAS G12C 9-mer (21, 29). Bear et al. also demonstrated that both 9-mer and 10-mer KRAS G12C peptides bind to HLA-A*03:01 and HLA-A*11:01. To our knowledge, our work describes the first TCR reactive against the KRAS G12C 9-mer presented on HLA-A*03:01 and HLA-A*11:01.

Previous studies have demonstrated the feasibility of targeting KRAS-mutant epitopes with TCRs against G12C (21), G12V (21, 27, 29–31), and G12D (21, 31–33) paving the way for novel therapeutic interventions in lung cancer. Some KRAS-specific TCRs have even demonstrated clinical efficacy (5, 34). Our study underscores the feasibility of targeting specific KRAS oncogenic mutations and contributes to the growing body of evidence supporting the feasibility of TCR-based cellular immunotherapy for lung cancer.

Our findings align with and extend observations from previous studies of the HLA-A3 superfamily. HLA-A*03:01 and HLA-A*11:01 share similar peptide-binding motifs and can present overlapping peptide repertoires. Structural and immunological studies have demonstrated that, despite comparable peptide presentation, subtle polymorphic differences between these alleles can impose strict TCR specificity (35, 36). In the context of viral immunity, identical peptides presented by HLA-A*03:01 and HLA-A*11:01 are often recognized by distinct T cell populations, with cross-recognition occurring only in limited cases mediated by individual TCRs (37). Large diversity of reactivity was observed in our KRAS-specific TCRs, including epitope degeneracy and strict HLA restriction (TCR-2 and TCR-5), epitope degeneracy with partial cross-HLA recognition (TCR-4), and robust HLA degeneracy with preserved specificity (TCR-1 and TCR-3).

A key finding of this study is that peptide degeneracy and HLA degeneracy are distinct properties. Several TCRs demonstrated substantial peptide degeneracy within a single HLA context and yet failed to recognize the same peptides presented on the alternate allele (TCR-2 and TCR-5, both TCRs against HLA-A*11:01). Conversely, certain TCRs, specifically against the G12V peptide, maintained their recognition hierarchy across both HLA-A*03:01 and HLA-A*11:01, suggesting an ability to tolerate structural variation in the peptide–HLA complex. This supports the concept that TCR recognition can sometimes be driven by conserved features of the peptide backbone or shared HLA contact residues, while in other cases remains highly sensitive to allele-specific conformational differences.

The alanine scan findings provided mechanistic insight into TCR degeneracy. Enhanced recognition of the G12A variant by TCR-1 may suggest that substitution at position 12 improves TCR engagement, potentially by altering peptide conformation. This is consistent with the ‘TCR plasticity’ notion that TCRs are inherently cross-reactive and can adjust to amino acid changes that preserve key structural features of the peptide–MHC complex (38). Our data place KRAS-specific TCRs within this notion, identifying receptors with both narrow and broad specificity profiles. An additional consideration for future studies is whether these TCRs can recognize alternative peptide lengths presented by HLA-A11:01. Previous work demonstrated that, for KRAS G12D, a 10-mer epitope elicited stronger immunogenic responses than the corresponding 9-mer peptide, suggesting that peptide length can substantially influence TCR recognition and antitumor reactivity (33). Therefore, evaluating the ability of our TCRs to recognize HLA-A*11:01-presented 10-mer variants of these epitopes may provide further insight into their functional breadth and could identify peptide formats with enhanced therapeutic potential.

From a translational perspective, these findings have important implications for TCR-based therapies targeting KRAS mutations. TCRs with cross-HLA recognition could expand patient eligibility across individuals expressing different alleles within the same superfamily, a long-standing goal in adoptive cell therapy. However, the observation that some TCRs exhibit enhanced reactivity to non-cognate peptides, a finding observed with TCRs against G12C (e.g., G12A preference over G12C in the case of TCR-1), underscores the need for comprehensive specificity profiling to mitigate potential off-target effects. Most importantly, all TCRs were also evaluated against the WT KRAS 9-mer peptide; increased reactivity was detected for all TCRs compared to WT which highlights a critical safety consideration for potential recognition of self-antigen.

In summary, our results demonstrate that KRAS-specific TCRs can exhibit diverse and occasionally unexpected cross-reactivity across both peptide variants and closely related HLA alleles. These findings reinforce the importance of empirically evaluating both peptide and HLA specificity for candidate therapeutic TCRs and suggest that cross-HLA targeting within the HLA-A3 superfamily is achievable. Overall, this study lays the foundation for future clinical development of engineered TCRs, which provides a novel and effective treatment strategy for patients with limited therapeutic options for lung cancer and other solid tumors.

## Supplementary Material

Supplementary Files

This is a list of supplementary files associated with this preprint. Click to download.

- ZhangandNassifRausseoCIITables.docx
- ZhangandNassifRausseoCIISupplementaryMaterial.pdf

Tables are available in the Supplementary Files section.

## Abbreviations

KRAS: Kirsten rat sarcoma viral oncogene homolog
TCR: T cell receptor
CAR: Chimeric antigen receptor
DC: Dendritic cell
NSCLC: Non-small cell lung cancer
GEMINI: Thoracic GEnomic Marker-guided Therapy INItiative
BLCA: Urothelial Bladder Carcinoma
BRCA: Breast Invasive Carcinoma
CESC: Cervical and endocervical cancers
CHOL: Cholangiocarcinoma
COAD: Colon Adenocarcinoma
DLBC: Diffuse Large B-cell Lymphoma
ESCA: Esophageal Carcinoma
GBM: Glioblastoma Multiforme
KIRC: Kidney Renal Clear Cell Carcinoma
KIRP: Kidney Renal Papillary Cell Carcinoma
LAML: Acute Myeloid Leukemia
LIHC: Liver Hepatocellular Carcinoma
LUAD: Lung adenocarcinoma
LUSC: Lung Squamous Cell Carcinoma
MESO: Mesothelioma
OV: Ovarian Serous Cystadenocarcinoma
PAAD: Pancreatic ductal adenocarcinoma
PRAD: Prostate Adenocarcinoma
READ: Rectum Adenocarcinoma
SKCM: Skin Cutaneous Melanoma
STAD: Stomach Adenocarcinoma
TGCT: Testicular Germ Cell Tumors
THCA: Thyroid Carcinoma
THYM: Thymoma
UCEC: Uterine corpus endometrial carcinoma
UCS: Uterine Carcinosarcoma
